# CT and ^18^F-FDG PET abnormalities in contacts with recent tuberculosis infections but negative chest X-ray

**DOI:** 10.1186/s13244-022-01255-y

**Published:** 2022-07-07

**Authors:** Soon Ho Yoon, Jin Mo Goo, Jae-Joon Yim, Takashi Yoshiyama, JoAnne L. Flynn

**Affiliations:** 1 Department of Radiology, Seoul National University College of Medicine, Seoul National University Hospital, 101 Daehak-ro, Jongno-gu, Seoul, 03080 Republic of Korea; 2 Institute of Radiation Medicine, Seoul National University Medical Research Center, Seoul, Republic of Korea; 3 Division of Pulmonary and Critical Care Medicine, Department of Internal Medicine, Seoul National University College of Medicine, Seoul, Republic of Korea; 4 Research Institute of Tuberculosis, Japan Anti-tuberculosis Association, Kiyose, Japan, Kiyose, Japan; 5 Department of Microbiology and Molecular Genetics and the Center for Vaccine Research, University of Pittsburgh School of Medicine, Pittsburgh, PA USA

**Keywords:** Tuberculosis, Incipient, Subclinical, Tomography (X-ray computed), Positron-emission tomography

## Abstract

Close contacts of individuals with pulmonary tuberculosis are at risk for tuberculosis infection and the development of active tuberculosis. In current contact investigations, immunologic tests (the tuberculin skin test and interferon-gamma release assay) and chest X-ray examinations are used to dichotomize contacts with *Mycobacterium tuberculosis* infections into those with active (X-ray abnormalities) versus latent tuberculosis (normal radiographs). This article is a critical review of computed tomographic (CT) and 18-fluorodeoxyglucose positron emission tomographic (PET) findings of incipient tuberculosis without X-ray abnormalities based on a systematic literature review of twenty-five publications. The CT and 18-fluorodeoxyglucose PET studies revealed minimal pauci-nodular infiltrations in the lung parenchyma and mediastinal lymph nodes abnormalities with metabolic uptake in approximately one-third of asymptomatic close contacts with negative chest radiographic and bacteriological/molecular results for active tuberculosis. Tuberculosis with minimal changes challenge the validity of simply dichotomizing cases of recent *M. tuberculosis* infections in contacts depending on the presence of X-ray abnormalities as the recent infections may spontaneously regress, remain stagnant, or progress to active tuberculosis in human and nonhuman primate studies. Whether contacts with tuberculosis with minimal changes are interpreted as having active tuberculosis or latent tuberculosis has clinical implications in terms of specific benefits and harms under the current contact management. Advanced imaging tools may help further stratify contacts intensely exposed to *M. tuberculosis* on a continuous spectrum from latent tuberculosis to incipient, subclinical and active tuberculosis. Identifying incipient tuberculosis would provide an opportunity for earlier and tailored treatment before active tuberculosis is established.

**Supplementary Information:**

The online version contains supplementary material available at 10.1186/s13244-022-01255-y.

## Key points

- Tuberculosis with minimal CT/PET changes challenges conventional radiographic dichotomization of active versus latent tuberculosis.
- Pauci-nodular lesions and LN abnormalities are CT and PET findings of incipient tuberculosis.
- CT and PET abnormalities in incipient tuberculosis may regress, stabilize, or progress.
- Identifying incipient tuberculosis may provide earlier and tailored treatment before active tuberculosis.

## Background

Tuberculosis (TB) is a major global public health problem. According to a 2016 study, latent infections with *Mycobacterium tuberculosis* (latent tuberculosis infections; LTBIs) affect approximately a quarter of the global population (1.7 billion people) [1]. In 2019, 10 million people developed active TB and 1.2 million died from the disease [2]; these numbers have declined slowly in recent years, but the coronavirus disease 2019 pandemic reversed this trend in 2020: Although new TB case reporting declined by 18%, TB-related deaths increased by one million [3]. In Europe, TB cases continued to decline between 2002 and 2019, but TB incidence varied considerably, from low rates in Western Europe to high rates in Eastern Europe [4]. Most active TB cases develop within 2 years of *M. tuberculosis* (Mtb) infection, with a small proportion of patients developing active TB later as a result of reactivation of LTBI [5]; This indicates that global TB cases are predominantly caused by recently transmitted Mtb infections [5].

Individuals in close physical contact with pulmonary TB patients are at risk for Mtb infection; thus, an investigation of close contacts is crucial for identifying cases of active TB early and preventing LBTI from progressing to active disease [6]. Indeed, contact investigations are effective for TB control and reducing TB-related mortality in both low-burden and high-burden settings [7, 8]. Contacts with active TB often present clinical symptoms and radiologic abnormalities and are diagnosed based on bacteriological (sputum smear or culture) or molecular (polymerase chain reaction assay) tests. In contrast, LTBI is diagnosed based on a positive tuberculin skin test (TST) or a T-cell-based (interferon-gamma release assay; IGRA) test but with no symptoms and radiologic abnormalities of active TB and negative bacteriological/molecular tests.

Radiologic abnormalities in LTBI are typically assessed using chest X-ray [6], which has limitations in visualizing small or subtle pulmonary lesions. Chest computed tomography (CT) scans can show the fine parenchymal lesions in active and healed TB [9, 10], and 18-fluorodeoxyglucose (^18^F-FDG) positron emission tomography (PET) scans can provide metabolic information beyond anatomic abnormalities [11, 12], contributing to an improved understanding of TB pathophysiology and manifestations. Healed TB refers to a remote Mtb infection, and the characteristic lung parenchymal CT findings of healed TB are well-established, including calcified or noncalcified granulomas, thin-wall cavities, cicatricial bronchiectasis, and end-stage lung destruction [10]. Healed TB typically has the maximum standardized uptake value below 1.5 on an ^18^F-FDG PET scan but may have a higher uptake in a minor portion, potentially raising a concern of TB reactivation [12]. In contrast, the CT and ^18^F-FDG PET findings of recent Mtb infections before the establishment of active disease have been sparsely reported. In this paper, we review the literature on the CT and ^18^F-FDG PET findings of recent Mtb infections before the establishment of active disease in humans with a representative case presentation, compare those findings with results from nonhuman primate models of Mtb infection, and discuss the implications and potential role of CT and ^18^F-FDG PET scans in a TB contact investigation.

## Main text

### Search strategy and study selection

One author (S.H.Y.; 7 years in experience of systematic reviews and meta-analyses, with more than 10 publications) conducted a systematic literature review of the OVID-MEDLINE and EMBASE databases to identify relevant articles. The author used a combination of keywords related to tuberculosis, CT, PET, and contact/outbreak/tracing/screening/latent up to October 25, 2021. This search was limited to literature with English titles and abstracts and was supplemented by screening the bibliographies of the retrieved articles and review articles.

We applied the following criteria for study selection: (a) prospective or retrospective human studies reporting adult or child contacts who were recently exposed to patients with pulmonary TB; (b) no symptoms, signs, or radiographic abnormalities suspicious for active TB; (c) a chest CT or ^18^F-FDG PET scan was performed in contact investigations. We excluded studies that used CT or PET scans (a) to evaluate contacts clinically or radiographically suspected of active TB in contact investigations, (b) to evaluate extrapulmonary TB in patients with active TB, (c) to identify remote TB infections (e.g., healed TB) in patients who were planned to receive immunosuppressive agents (e.g., for rheumatic diseases or transplantation), and (d) to screen for incidental TB (e.g., pre-employment or health worker screening) regardless of recent TB exposure.

### Included studies

Of 1638 publications identified in the initial database search, 25 articles studies have applied chest CT, ^18^F-FDG PET/CT, or magnetic resonance imaging (MRI) to evaluate asymptomatic close contacts with pulmonary TB patients and normal radiographs (Table 1) (Additional file 1: Fig. 1): 21 CT studies [13–33], two ^18^F-FDG PET/CT studies [34, 35], and two PET/MR studies [36, 37]. Seventeen of the 25 studies were conducted in Asia, followed by Europe. The median number of contacts undergoing advanced imaging was relatively small, at 32. Twelve studies examined children to adolescents [13–16, 18, 21, 23–25, 27, 28, 30], while the other studies included adults. One study was conducted on contacts infected with HIV [35], while the other 24 studies were conducted in immunocompetent contacts. Four studies partly [25, 29] or entirely [23, 26] included close contacts with drug-resistant TB. Bacterial and molecular testing with sputum or other specimens (e.g., gastric aspirates) generally yielded a low positivity rate in contacts with minimal CT or PET abnormalities, reflecting a low bacillary burden (Table 1).

**Table 1:** Summary of studies using chest CT, ^18^F-FDG PET/CT, or MRI in asymptomatic contacts recently exposed to TB with normal radiographs

First author	Year	Country	No. of contacts* (age range)	No. of contacts with CT or PET abnormalities			Bacterial/molecular Testing		Anti-TB treatment	Follow-up without anti-TB treatment/Developing TB during follow-up
				Any	Lung** parenchyma	Mediastinal LN	Tested	Positive		
*Chest CT*										
Delacourt [13]	1993	France	15 (0–10 s)	9 [60%]	0 [0%]	9 [60%]	Not separable		Not described	Not described
Duran [14]	1996	Spain	22 (0–10 s)	14 [64%]	4 [18%]	14 [64%]	8	4 [50%]	Not described	Not described
Katakura [15]	1999	Japan	4 (0 s)	4 [100%]	3 [75%]	1 [25%]	4	0 [0%]	4 [100%]	None
Baghaie [16]	2005	Iran	64 (4–14)	25 [39%]	Not separable		Not separable		Not described	Not described
Yoshiyama [17]	2008	Japan	21 (20–40 s)	7 [33%]	7 [33%]	0 [0%]	7	1 [14%]	7 [100%]	None
Lew [18]	2009	Korea	46 (16–20)	12 [26%]	12 [26%]	0 [0%]	11	0 [0%]	11 [92%]	1^†^/1
Lee [19]	2010	Korea	39 (20 s)	9 [23%]^‡^	9 [23%]	0 [0%]	9	3 [33%]	9 [100%]	None
Hirama [20]^‡^^†^	2011	Japan	34 (adults)	4 [12%]	4 [12%]	0 [0%]	Not described		4 [100%]	None
Garrido [21]	2012	Spain	11 (< 4 years)	9 [81%]	7 [64%]	9[81%]	11	1 [9%]	11 [100%]	None
Fujikawa [22]	2014	Japan	110 (20–50 s)	12 [11%]	12 [11%]	0 [0%]	12	3 [25%]	12 [100%]	None
Catho [23] ^‡‡^	2015	France	5 (child)	1 [20%]	1 [20%]	1 [20%]	1	1 [100%]	1 [100%]	None
Lu [24]	2016	China	27 (10 s)	6 [22%]	6 [22%]	0 [0%]	6	0 [0%]	6 [100%]	None
Ziemele [25]	2017	Latvia	Unknown (0–17)	145 [unknown]	Not separable		145	7 [5%]	145 [100%]	None
Lee [26]^‡‡†^	2017	Korea	6 (30 s)	2 [33%]	2 [33%]	0 [0%]	2	0 [0%]	0 [0%]	2/1
Shimizu [27]	2017	Japan	52 (0–10 s)	10 [19%]	Not separable		10	0 [0%]	Not separable	None
Moreno-Ballester [28]	2018	Spain	52 (0–10 s)	27 [52%]	17 [33%]	27 [52%]	Not described		27 [52%]	None
Yoshiyama [29]	2019	Japan	229 (10–80 s)	24 [10%]	24 [10%]	0 [0%]	24	7 [29%]	23 [96%]	1/follow-up loss at 3 mo
Zhou [30]	2019	China	59 (10 s)	51 [86%]	Not separable		Not separable		35 [69%]	15***/1
Yoon [31] ^‡‡‡^	2020	Korea	17 (50–70 s)	14 [82%]	14 [82%]	0 [0%]	Not described		0 [0%]	14/0
Wang [32]	2020	China	135 (10–60 s)	4 [3%]	Not separable		4	0 [0%]	4 [100%]	None
Mok [33]	2021	Korea	72 (adults)	14 [19%]	14 [19%]	0 [0%]	14	1 [7%]	0 [0%]	14***/1
^*18*^*F-FDG PET/CT*										
Ghesani [34]	2014	USA	5 (20–40 s)	3 [60%]^‡‡‡^^†^	0 [0%]	3 [60%]	Not described		0 [0%]	3***/0
Esmail [35]^‡‡‡‡^	2016	UK	35 (20–30 s)	16 [46%]	10 [29%]	16 [46%]	16	0 [0%]	0 [0%]	16***/4
^*18*^*F-FDG PET/MRI*										
Molton [36]	2019	Singapore	30 (0–50 s)	9 [30%]	4 [13%]	6 [20%]	Not described		0 [0%]	9***/0
Naftalin [37]	2020	Singapore	3 (20–50 s)	2 [67%]	2 [67%]	2 [67%]	Not described		Not described	Not described

TB —tuberculosis; CT —computed tomography; ^18^F-FDG— 18-fluorodeoxyglucose; PET —positron emission tomography; MRI —magnetic resonance imaging

Data in parentheses and brackets indicate age range and percentage, respectively

*Contacts having symptoms or abnormal X-ray findings were not counted

**CT abnormalities are pauci-nodular infiltrations in the lung parenchyma, not including healed TB sequelae

***LTBI treatment was applied to in part or entire contacts during the follow-up

^†^One contact with a 5-mm nodule developed active TB during follow-up. It was not clearly described whether the contact received LTBI treatment

^‡^Seven of nine contacts showing CT abnormalities were reported to have cough or sputum

^‡†^Two of four patients treated with anti-TB medication turned out to have been misdiagnosed after the completion of treatment

^‡‡^Baseline CT was normal, but follow-up CT after 3 months revealed two consolidative lesions in the right inferior lobe with mediastinal lymphadenopathy

^‡‡†^One of the two contacts had a transient increase in pauci-nodular lesions, followed by a decrease during 1 year

^‡‡‡^Small noncalcified nodules spontaneously regressed in 2 of 14 patients after 1 year, whereas small noncalcified nodules remained stable in 12 of 14 patients

^‡‡‡†^We did not count one contact having ^18^F-PET/CT findings of healed TB

^‡‡‡‡^Eight of 10 contacts with subclinical lung abnormalities and 8 of 25 contacts without the abnormalities had metabolic uptakes in mediastinal LNs

The median prevalence of minimal CT and/or PET abnormalities was 33.0%, but the range varied substantially from 3 to 100%. The wide range of prevalence may have resulted from differences in study populations, (including how to define close contacts having a risk for recent TB infections), the exposure intensity and infectivity of the index pulmonary TB case (more intensive exposures can lead to a higher prevalence), imaging modalities (lung parenchymal abnormalities on non-enhanced chest CT versus lung parenchymal and mediastinal and/or extrapulmonary abnormalities on contrast-enhanced chest CT or ^18^F-FDG PET-CT or MR), and definitional criteria for interpreting radiologic or metabolic abnormalities on imaging modalities.

### CT and PET findings of recent TB infections in humans

**Fig. 1:**
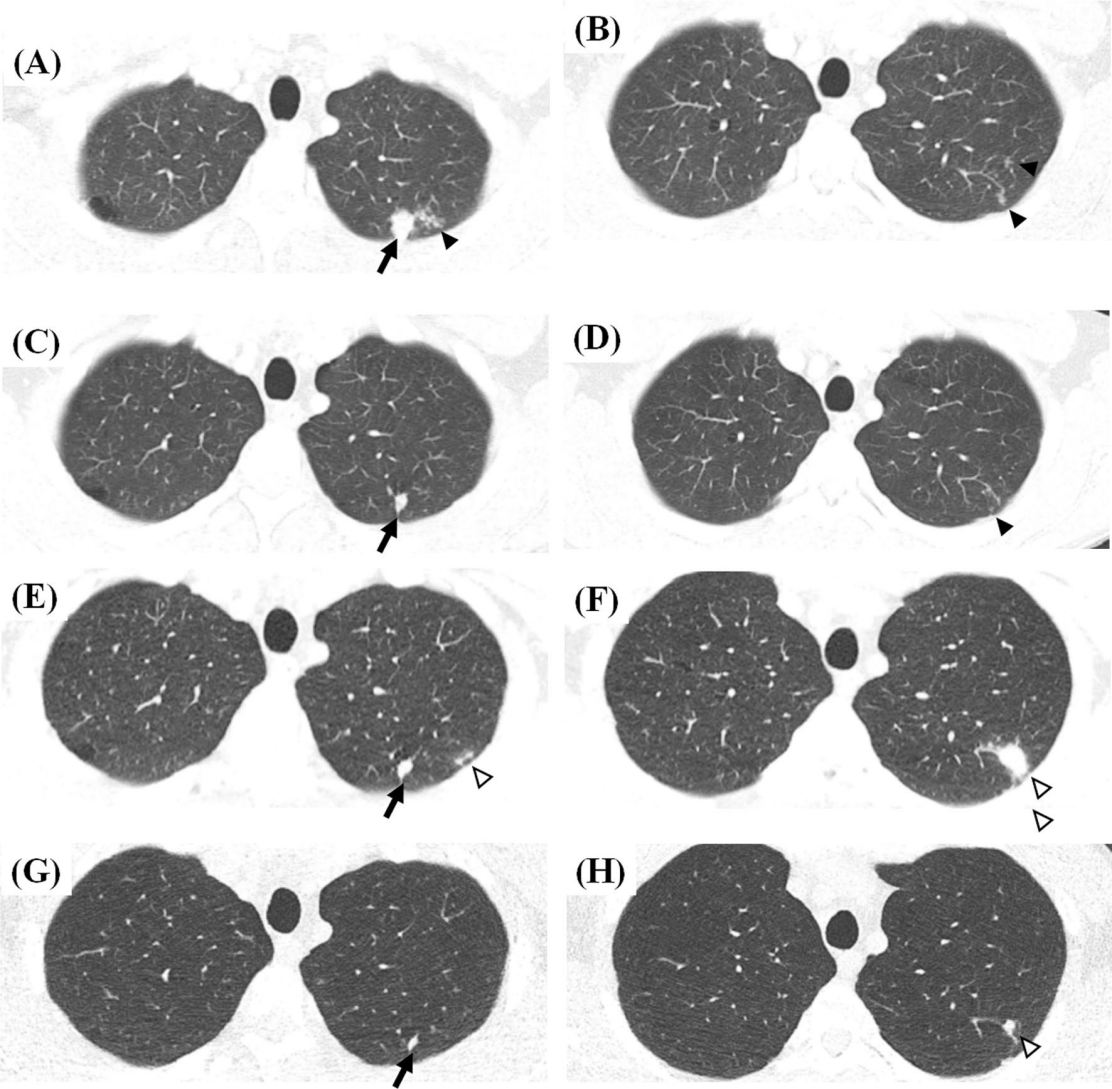
Representative CT images showing dynamics of recent tuberculosis infection in a 33-year-old male contact of a patient with infectious multi-drug resistant tuberculosis. **a**, **b** Baseline CT images show a nodular consolidation (black arrow) and micronodules (black arrowhead) in two secondary pulmonary lobules in the apicoposterior segment of the left upper lobe (**a**). Another minimal pauci-nodular infiltration is noted in the adjacent subsegmental lobules (black arrowhead) (**b**). Bacteriologic/molecular tests of bronchoscopic alveolar lavage fluid were negative. The patient was observed without treatment. **c**, **d** Decreases in the previous pauci-nodular infiltrations are shown on follow-up CT images 14 months after the baseline CT examination. Sputum smear and culture were negative. **e**, **f** Eighteen-month follow-up CT images show new nodular consolidation and adjacent pauci-nodular infiltrations (white arrowheads) from one of the shrunken nodules (**e**, **f**), while another nodule remains stable (black arrow) (**e**; variable changes among lesions within a host). The close contact denied any symptoms and signs. Bacteriologic/molecular tests of bronchoscopic alveolar lavage fluid were negative. However, radiologic progression was regarded as indicating active tuberculosis, leading to the subsequent initiation of anti-multidrug resistant tuberculosis treatment. **g**, **h** Follow-up CT images 5 months after treatment show decreases in the previous nodular lesions with a few residual small nodules (black arrow and white arrowhead)

Delacourt et al. [13] first used chest CT scans to evaluate children suspected of having been recently infected with TB without clinical and radiographic abnormalities. Among 15 children, nine (60%) had enlarged right paratracheal and hilar lymph nodes (LNs) without lung parenchymal lesions. Similarly, Durán et al. [14] reported enlarged mediastinal LNs (14 of 22 children) and peripheral lung infiltrations (4 of 22 children) in asymptomatic children with TB infections and normal radiographs, but there were no detailed descriptions of the parenchymal infiltrations. Katakura et al. [15] and Yoshiyama et al. [17] elaborated on morphologic findings of lung parenchymal abnormalities on CT images. Most CT abnormalities were a few small nodules smaller than 1 cm or micronodular infiltrations in a limited number of peripheral secondary pulmonary lobules, preferentially in the upper lobes (Fig. 1a, b). Similar morphologic descriptions of CT or MRI findings could be found in some other studies that provided sufficient information on morphologic characteristics [20, 21, 26, 29, 31].

PET imaging visualized increased metabolic uptake in the lung parenchyma with or without identifiable lesions, and those findings were frequently accompanied by increased metabolic uptake in LNs confined to the hilum and mediastinum [34–37]. Interestingly, the involved mediastinal LNs tended not to be sufficiently enlarged to be detected on CT in adults, while children frequently had enlarged mediastinal LNs with or without necrosis [13–15, 21, 23, 28].

### Follow-up of CT and PET findings of recent TB infections in humans

When CT or PET abnormalities were identified, most of the studies initiated full regimens of anti-TB medication solely based on radiologic or metabolic abnormalities without bacteriological/molecular test positivity for active TB. Contacts with minimal CT or PET abnormalities were treated successfully, including a treatment success rate (successful treatment completion with or without bacteriological evidence of success [38]) of 100% in twenty-two contacts younger than 18 years exposed to multi- or extensively drug-resistant TB [25]. There were two similarly treated contacts exposed to multi-drug resistant TB in the literature [23, 26].

It is rarely possible to prospectively monitor how untreated active pulmonary TB first manifests after infection and changes at an incipient stage in humans. Observing patients newly infected with Mtb without providing treatment may be controversial, as LTBI treatment effectively prevents progression to active disease in drug-sensitive or multidrug-resistant TB [39], although close regular monitoring can allow early detection of progressing active disease without LTBI treatment. Likewise, it would be unethical to acquire lung tissues for investigational purposes in these infected but asymptomatic patients. Eight studies reported follow-up data of minimal radiologic and metabolic abnormalities without anti-TB treatment [26, 31, 34, 35]: six studies applied LTBI treatment to in part or entire study populations [18, 30, 33–36], while two studies reported findings in patients who did not receive any treatment [26, 31].

The first follow-up observations of minimal abnormalities were made using ^18^F-FDG PET scans in 35 HIV-infected contacts who received LTBI treatment using isoniazid monotherapy [35]. Among them, 10 contacts had normal scans and 25 had baseline CT abnormalities, including 9 with infiltrates and/or fibrotic scars, 1 with hematogenous TB spread, and 15 with nonspecific discrete nodules. The authors regarded the 10 contacts with pulmonary infiltrates or hematogenous TB spread in the lung parenchyma as having minimal abnormalities. Hypermetabolism in mediastinal LNs was noted in 10 contacts with minimal abnormalities and 6 of 25 contacts without abnormalities. During a 6-month follow-up, 4 of these 10 contacts progressed to have culture-proven (*n* = 2) or clinically deteriorated (*n* = 2) TB, whereas TB did not develop in the remaining 25 contacts. With the exception of 4 patients who developed TB disease, 6-month follow-up PET/CT scans revealed that isoniazid monotherapy decreased pre-existing CT abnormalities in all six contacts with minimal abnormalities and metabolic LN uptake in 1 contact without parenchymal abnormalities [35]. Similarly, asymptomatic hypermetabolism of mediastinal LNs based on ^18^F-PET/CT findings [34] or pauci-nodular lesions [30, 33] decreased with LTBI treatment.

Another follow-up study performed submillisievert CT scans in 6 immunocompetent close contacts with multidrug-resistant TB [26] when standardized LTBI treatment was not well established. All the contacts were asymptomatic with normal radiographs, and half of the contacts were positive for both TST and IGRA. CT scans showed a minimal extent of small noncalcified nodular infiltrations in two contacts, but they had contradictory courses: one contact developed active multidrug-resistant TB shortly thereafter, while the lesion in the other contact regressed spontaneously. A few similar cases with minimal abnormalities progressing into active TB were reported in contacts who denied LTBI treatment [30, 33]. In the contrary, spontaneous regression of noncalcified nodules on CT scans was also noted in one prospective investigation of seventeen close contacts without LTBI under close regular monitoring [31].

**Fig. 2:**
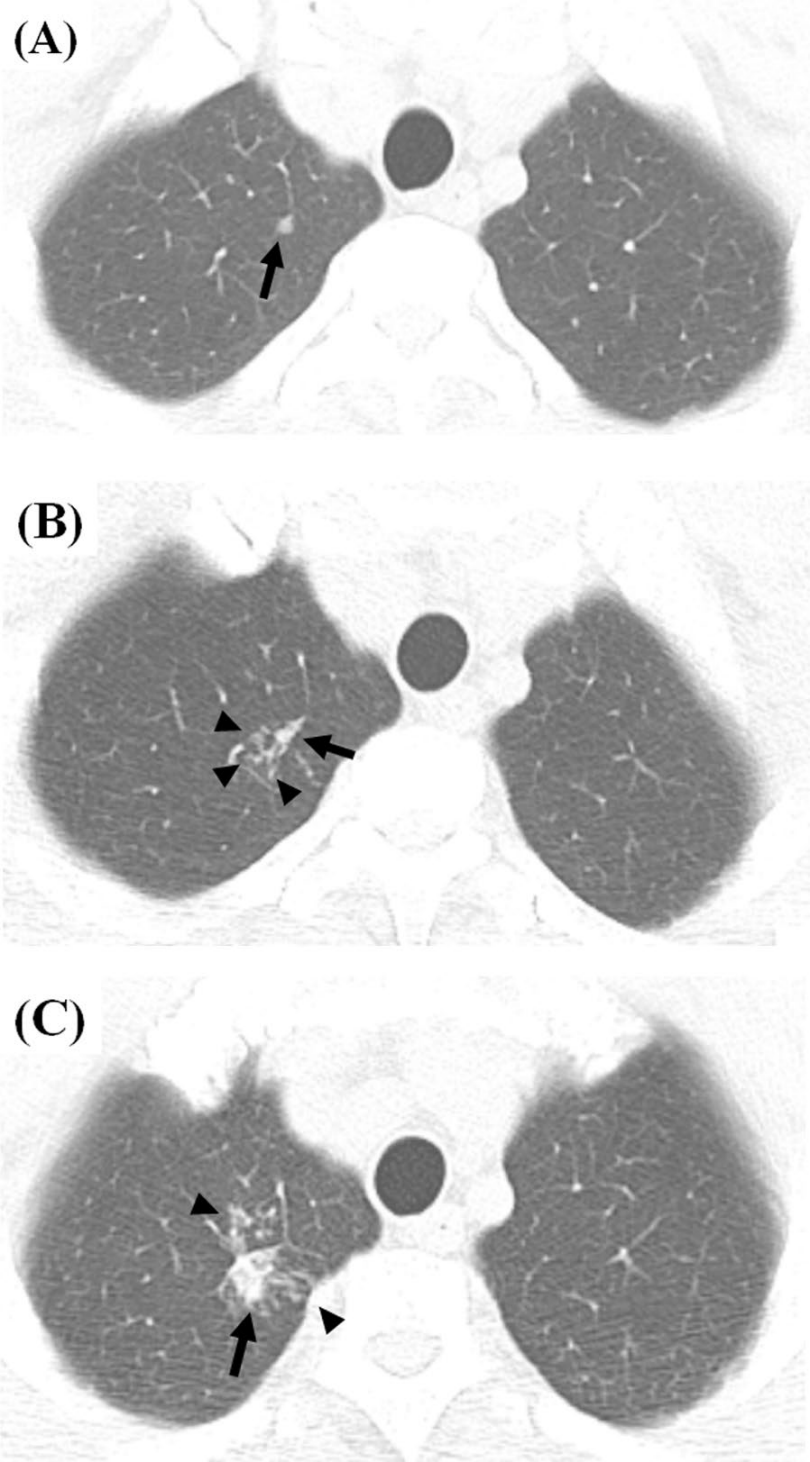
Representative CT images showing incipient TB progressing into active tuberculosis disease in a 52-year-old healthy male. **a** A screening CT image shows a small single incidental nodule in the right upper lobe (black arrow). **b** A follow-up CT image 20 months later reveals a few additional micronodules (black arrowheads) in a secondary pulmonary lobule around the small pre-existing nodule (black arrow). The patient denied any symptoms and signs and had negative sputum smear and culture tests. **c** The next follow-up CT image 24 months later shows the increasing size and number of the nodule (black arrow) and micronodules (black arrowheads) in the right upper lobe. The patient still denied any symptoms and signs. The sputum smear was negative, and *Mycobacterium tuberculosis* was confirmed on sputum culture. The patient had a 6-month standard anti-tuberculosis medication and healed without sequelae

**Fig. 3:**
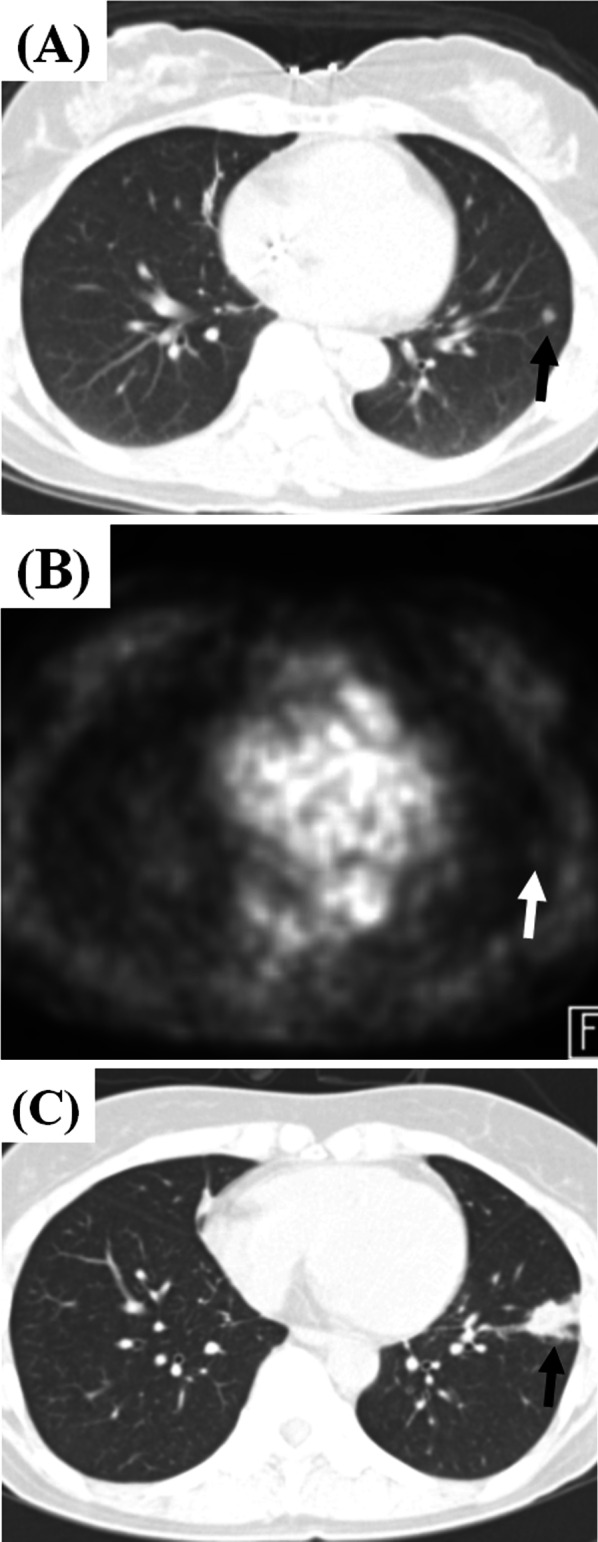
Representative CT and 18-fluorodeoxyglucose PET images showing incipient TB progressing into active tuberculosis disease in a 60-year-old female with malignancy. **a,** **b** CT and PET images for metastasis work-up shows a small single incidental nodule with subtle hypermetabolism in the left lobe lobe (black arrow). There was no metabolic uptake in mediastinal lymph nodes. (**c**) A follow-up CT image 6 months later reveals the increasing size of the nodule with micronodular infiltration (black arrow). The patient still denied any symptoms and signs. The sputum smear and culture was negative for *Mycobacterium tuberculosis*. The specimen of percutaneous transthoracic lung biopsy turned to be chronic granulomatous inflammation with necrosis, consistent with active tuberculosis disease

As an example of the dynamic changes in minimal radiologic abnormalities, we present the case of a 33-year-old male close contact (Fig. 1), The contact had worked in the same office with a patient with multi-drug resistant TB for 1 year and had smoked cigarettes for 10 years without any comorbidities. He denied any symptoms and signs of active TB. In a contact investigation, TST and IGRA were positive, and sputum smear and culture were negative. Chest CT was conducted to evaluate an equivocal small nodular opacity on a chest radiograph. Baseline CT images (Fig. 1a, b) showed a nodular consolidation and a few micronodules in the apicoposterior segment of the left upper lobe. Bronchoscopy was performed, and bacteriologic/molecular tests of bronchoscopic alveolar lavage fluid were negative. The patient was observed without treatment. On the first follow-up CT scan (Fig. 1c, d; 14 months after baseline), the previous pauci-nodular infiltrations decreased, but a residual nodule was present. He visited our hospital for a second opinion, and the second set of follow-up CT images (Fig. 1e, f; 18 months after baseline) showed new nodular consolidation and adjacent pauci-nodular infiltrations from one of the shrunken nodules, while another nodule remained stable, indicating that lesions within a single host may show variable changes. He still denied any symptoms and signs. Bacteriologic/molecular tests of the sputum and bronchoscopic alveolar lavage fluid were negative. However, radiologic progression was regarded as indicating active TB, leading to the subsequent initiation of anti-multidrug resistant TB treatment. The third set of follow-up CT images (Fig. 1g, h; 5 months after treatment and 23 months after baseline) showed reductions in the previous nodular lesions with a few residual small nodules, radiologically similar to healed TB with negligible sequelae. Two additional cases (Figs. 2 and 3) also illustrate that active TB developed without apparent signs or symptoms or positive microbiologic tests, but CT detected the changes of incipient TB, indicating progression to active disease.

In summary, CT and PET scans showed incipient minimal pauci-nodular infiltration in the lung parenchyma with metabolic uptake in the infiltrate and mediastinal LNs in approximately one-third of asymptomatic close contacts with negative chest radiographic and bacteriological/molecular results for active TB. Despite limited observations, these contacts may show various changes during follow-up without treatment. However, the considerable limitations in comprehensively elucidating the natural course of untreated minimal radiologic or metabolic abnormalities make it necessary to refer to the natural course of Mtb infections in nonhuman primates.

### Histopathologic, CT, and PET Findings of recent TB infections in nonhuman primates

One of the major areas of progress in TB animal models has been the establishment of LTBI in cynomolgus macaques [40]. Cynomolgus macaques are 40- to 50-cm primates that are relatively resistant to *M. tuberculosis* compared to other species used for TB research [41] in that these macaques develop the full range of human Mtb infection outcomes, from LTBI to severe active disease. A bronchoscopic instillation of low-dose (< 10^2^ colony forming unit) virulent *M. tuberculosis* in cynomolgus macaques results in an approximately even distribution of active TB and LTBI [41]. Cynomolgus macaques with LTBI were found to have no clinical signs of disease for at least 6 months after infection, with normal chest radiographs. Necropsies of macaques classified as LTBI revealed a limited number of small granulomas in the lung parenchyma with corresponding mediastinal lymphadenopathy, including a granuloma in a mediastinal LN, in some of the animals [42]. The granulomas showed a histologic spectrum from caseous granulomas with mineralization and/or collagenous materials to non-necrotizing sclerotic granulomas and completely fibrotic granulomas with an admixture of amorphous calcification and fibrotic tissues. Although it is difficult to follow infected macaques for long periods of time due to space constraints in biosafety level3 facilities, macaques with LTBI can be stable up to 20 months after infection. However, some macaques with LBTI progressed to active TB during follow-up [43]. Longitudinal monitoring of LTBI progression using ^18^F-FDG PET/CT scans showed varying fates of granulomas when over the course of Mtb infection [44]. Each lung parenchymal granuloma in LTBIs was typically initiated by a single bacterium and could spontaneously regress, persist, progress, or newly appear in a single infected host. Thoracic LN involvement can also be tracked in macaques by PET/CT imaging [45]. One or more mediastinal LNs become apparent by PET/CT in macaques within 4 weeks of infection and granulomas are often found in LNs at necropsy at multiple time points. In fact, LNs are a site for bacterial persistence in macaques [46].

In cynomolgus macaque models, recent Mtb infection may cause a few subcentimeter granulomas beyond the radiographic resolution and involve thoracic LNs, and the granulomas show lesion-by-lesion patterns of dynamic change, including spontaneous resolution, stabilization, and progression to active disease.

### Interpretation of minimal CT or PET abnormalities in current contact investigations

**Fig. 4:**
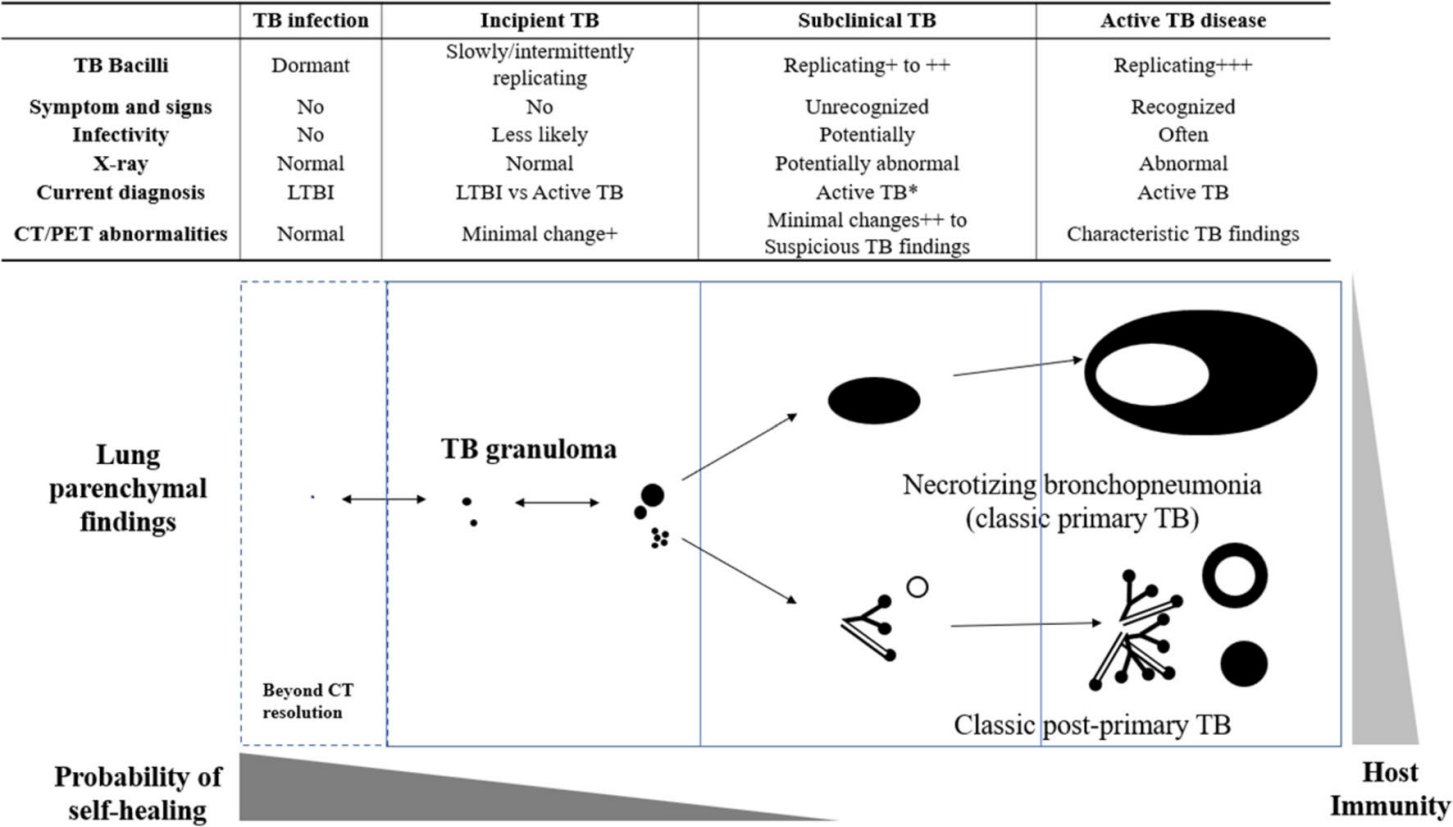
Modern understanding of the tuberculosis disease spectrum, current diagnosis, and CT and.^18^F-FDG PET abnormalities. *Close contacts to tuberculous patients with X-ray abnormalities are regarded as patients with active disease

TB is a continuum encompassing LTBI, incipient, subclinical TB, and active TB [47, 48] (Fig. 4), and subclinical TB can have X-ray abnormalities or microbiological evidence of active, viable *M. tuberculosis* without symptoms suggestive of active TB disease [47]. Minimal CT or PET abnormalities in asymptomatic close contacts without X-ray abnormalities correspond to incipient TB, which slowly and intermittently replicates. In current contact investigations, the minimal CT or PET abnormalities can be interpreted as either foreshadowing active TB or undetermined latent TB. The former interpretation necessitates anti-TB medication, whereas the latter interpretation requires LTBI management. In the latter interpretation, clinical or radiologic changes during follow-up can differentiate progressing active TB from stagnant or regressing latent TB.

Both interpretations have specific benefits and harms (Table 2). Labeling patients as having active TB, followed by anti-TB treatment, can prevent early undetermined cases from progressing to active TB and curtail TB transmission. However, active TB could be over-diagnosed [49, 50], and LTBI patients may receive unnecessary anti-TB treatment, even though patients may have a chance of spontaneous resolution with LTBI treatment. On the contrary, labeling patients as having latent TB, followed by monitoring, may miss the chance for earlier treatment and blockage of subsequent TB transmission or place contacts at risk of acquiring resistance to LTBI drugs [51] (although the risk is very low), if cases progress to active TB. Classifying these cases as latent TB rather than active TB seems more conventional and consistent with the results of the current contact investigations using X-ray examinations. However, categorizing these cases as active disease may help control TB outbreaks in crowded places such as prisons, military barracks, or quarantine asylums where TB can be transmitted massively in a short period and should be controlled without delay.

**Table 2:** Benefits and harms of interpreting minimal CT/PET abnormalities as active/latent disease in asymptomatic close contacts

	Benefits	Harms
Labeling as active disease with anti-TB treatment	Minimizing progression to active TB and potential subsequent TB transmission	Anti-TB medication without bacteriological/molecular TB evidence
	Minimizing the risk of acquired resistance to LTBI drugs	Unnecessary anti-TB medication regimens that is longer and has more side effects than LTBI medication regimens^†^
		Overestimation of TB outbreaks by inflating the number of active TB cases
Labeling as latent disease with follow-up	Giving a chance for self-healing with LTBI management	Risk for progression to infectious TB and potential subsequent TB transmission while observing^‡^
	Estimation of the number of active TB cases in outbreaks based on bacteriological/molecular TB evidence	Chance of acquired resistance to LTBI drugs^‡†^
	Agreement with the results of conventional contact investigation using X-ray examinations	Follow-up may increase radiation exposure to patients^‡‡^

CT —computed tomography; PET— positron emission tomography; TB —tuberculosis; LTBI —latent tuberculosis infection

^†^A recent LTBI guideline recommends 3- to 4-month rifamycin or rifampin-based regimens instead of 6- to 9-month isoniazid monotherapy [51]

^‡^The risk may vary depending on the follow-up interval and preventive measures such as mask-wearing

^‡†^The use of isoniazid and rifampicin in LTBI treatment did not significantly increase the chance of acquired resistance to the corresponding drugs in meta-analyses [82, 83]

^‡‡^X-ray examinations or computed tomographic imaging can be used to assess radiologic changes

### Potential role of CT and PET imaging in TB contact investigations as continuum disease

**Fig. 5:**
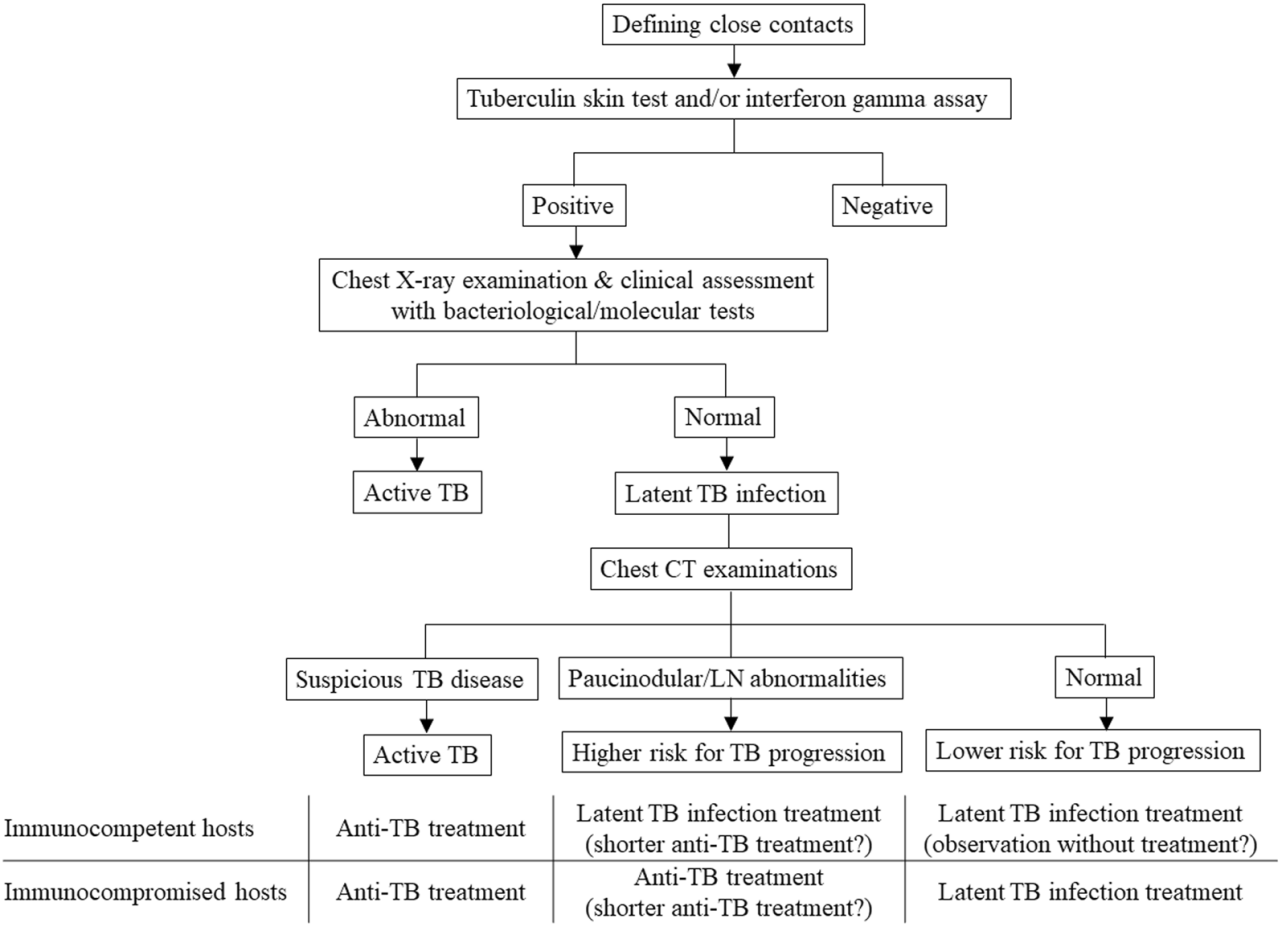
Potential role of chest CT scans in TB contact investigation

A potential advantage of CT and ^18^F-FDG PET imaging in TB contact investigations is that these modalities may enable earlier detection of active TB (i.e., before it becomes full-blown) in patients with normal radiographs requiring anti-TB medication and further stratification of contacts with recent LTBI who are at the higher risk for developing active TB (minimal abnormalities) from those who are less likely to develop (no abnormalities) (Fig. 5).

The recommended US Centers for Disease Control and Prevention treatment regimens for drug-sensitive LTBIs include 3-month once-weekly isoniazid plus rifapentine, 4-month daily rifampin, and 3-month isoniazid plus rifampin with alternative options of daily isoniazid for 6 or 9 months [52]. All three regimens showed odd ratios of 0.25–0.36 against developing active TB compared to no treatment [52]. Multidrug-resistant LTBI treatment also provides a relative risk reduction against developing active TB by 90%, although the LTBI treatment regimen has not been standardized [53].

LTBI treatment is initiated for close contacts based on the result of TST and/or IGRA positivity. The positive predictive values of TST and IGRA for developing active TB are limited to an average of 2.7% and 1.5% over a 2-year period after TB infection, respectively [54], suggesting that narrower criteria are needed to identify contacts at high risk for developing TB. Recently introduced whole-blood transcriptional signatures are a promising tool to improve prediction, but their positive predictive values were 6.8–9.4% over 2 years and 11.2–14.4% over 3 months based on a pre-test probability of 2% [55]. The 2-year positive predictive values did not meet the World Health Organization (WHO) target for predicting progression from TB infection to active disease, although the 3-month result exceeded the minimum of the WHO target [56].

Currently, radiographically-negative asymptomatic cases with incipient TB receive LTBI treatment, as they are classified as having latent TB due to normal X-ray findings. In a small series of HIV-infected contacts, TB progression exclusively occurred in contacts with minimal CT or PET abnormalities [35]. Greater bacterial burdens place patients at a higher risk for progression, and the size and metabolic uptake of abnormalities can reflect the quantity of bacilli and associated inflammatory burden [57, 58]. Taken together, close contacts with minimal CT or PET abnormalities may have a higher risk for TB progression than those without such abnormalities.

Treatment initiation for incipient TB with a low bacillary burden may be a potential way to improve the treatment outcomes of TB [59, 60] and reduce the probability of acquiring drug resistance [61]. This may be particularly beneficial for close contacts with multi- or extensively drug-resistant TB that needs relatively long treatment but is less likely to respond to treatment when active TB is established [25, 62]. Since these minimal CT and PET abnormalities pathologically occupy a position between LTBI and active TB, treatment may also be tailored by introducing a shorter abbreviated regimen, as indicated by the recent results of the SHINE (Shorter Treatment for Minimal Tuberculosis in Children) trial reporting non-inferiority of 4 months of treatment to the standard 6-month treatment regimen for non-severe TB [63].

CT or PET imaging in TB contact investigations may further benefit immunocompromised contacts (i.e., those with HIV) or contacts suspected of having intense TB exposures [64] who are at higher risk for TB infection and active disease [65]. Immunocompromised hosts with active TB can have normal radiographs in up to 10–40% of cases, and often have mediastinal lymphadenopathy or extrapulmonary diseases that can only be evaluated to a limited degree using chest radiographs [66]. Furthermore, miliary TB relatively frequently develops in immunocompromised hosts, but 59 to 69% of miliary TB cases are detectable on chest radiographs [67]. If minimal CT or PET abnormalities are shown in immunocompromised contacts, labeling these cases as active disease may be more suitable for immunocompromised or immunosuppressed contacts, since that they have a higher chance of TB progression [6].

### Practical considerations of CT and PET imaging in TB contact investigations

Chest CT and ^18^F-FDG PET scans are advanced imaging tools to evaluate recent TB infections, but before use, practitioners should rigorously assess whether the use of advanced imaging tools will add value to the contact investigation. The current contact investigation and LTBI treatment methods are effective for TB control, and advanced imaging tools will be worthless in most situations if the tools are used indiscriminately. There are two fundamental points to consider: (1) the risk of TB infection and developing active TB and (2) the probability of active TB manifesting as normal radiographs. If the risk is not sufficiently intense (e.g., in a mass screening context), most examinations will not additionally depict changes from incipient TB to active TB beyond what is portrayed by X-ray examinations, as TB disease infrequently occurs and progresses [29, 50, 68, 69]. TB more frequently manifests with normal radiographs in immunocompromised contacts or children. Esmail’s study on HIV contacts using ^18^F-FDG PET/CT is a representative example of how advanced imaging can add value to contact investigations [35].

Each modality has its own advantages and disadvantages (Table 3). Chest CT radiation is a matter of concern, but it can be minimized to be comparable to the doses of chest frontal and lateral radiographs for evaluating TB lesions [26, 31, 70]. As an alternative imaging modality, ^18^F-FDG PET may be considered, but it can deliver a greater radiation dose than chest CT, potentially raising the risk of cancer [71, 72]. Chest CT scans are much more broadly implemented, are less expensive, faster obtainable, and may be more cost-effective [73] than ^18^F-FDG PET scans. Chest CT scans can detect incipient pulmonary TB and intrathoracic extrapulmonary TB (TB pleurisy and enlarged TB lymphadenitis), but cannot cover extrathoracic TB diseases, which is evaluable by ^18^F-FDG PET scans [74]. Nevertheless, incipient TB primarily manifests in the thorax [75], and even in cases with extrapulmonary TB, TB pleurisy comprises more than half of extrapulmonary TB [76]. Therefore, chest CT scans are a more reasonable and practical modality in most contact investigations than ^18^F-FDG PET scans may be considered in contacts at risk for a higher chance of extrapulmonary TB, such as HIV-infected persons with low CD4 counts [77].

**Table 3:** Advantages and disadvantages of advanced imaging modalities for assessing recent TB infections before active disease

	Advantages	Disadvantages
Chest CT	Widely implemented	Evaluation of extrapulmonary TB manifestations
	Radiation dose comparable to chest radiographs	Evaluation of TB lesions without morphologic changes (e.g., LNs)
	Quick acquisition in a single breath-hold	
	Relatively affordable	
^18^F-FDG PET	Metabolic information is quantifiable	Expensive
	Extrapulmonary lesions are evaluable	Radiation dose is higher than chest CT
	TB lesions without morphologic changes are detectable	Long acquisition time
	Respiratory motions may affect image quality	Limitedly accessible in resource-constraint settings
		Detection of small pulmonary lesions may be limited*

TB —tuberculosis; CT —computed tomography; ^18^F-FDG —18-fluorodeoxyglucose; PET —positron emission tomography; MR —magnetic resonance; LN— lymph node

*Detection of small pulmonary lesions may be limited on a PET/MR scan

In most of the included studies, radiologists evaluated radiologic or metabolic abnormalities subjectively without pre-specified definition (Additional file 1: Table 1), demanding the necessity for standardized definitional criteria. CT size criteria for assessing enlarged mediastinal LNs in children varied in the relevant studies [78], and LNs with similar metabolic uptake on an ^18^F-FDG PET scan may be less specific in TB-endemic areas than in non-TB-endemic areas [79]. Furthermore, CT or PET findings of incipient TB are not specific for TB in 100%, demanding the exclusion of other alternative diagnoses [80, 81]. Lastly, it has not been established which findings should be called minimal CT or ^18^F-FDG PET parenchymal abnormalities with an undetermined fate and which findings are suggestive of irreversible progression to active TB. At a minimum, a thick-walled cavity is a hallmark of active TB [82], although a thin-walled cavity may present in both active and healed TB [10]. According to the modern understanding of TB pathology in humans [83], asymptomatic obstructive bronchioles and associated lobular pneumonia seem to be a key process in establishing active TB, but spontaneous resolution occurs in 95% of cases before caseation, while the remaining 5% of cases evolve into caseous pneumonia, the irreversible tipping point. Caseous obstruction of bronchioles manifests as the tree-in-bud sign [84, 85]; therefore, the overt tree-in-bud sign on CT images also may be regarded as indicating active TB.

We suggest that minimal parenchymal CT abnormalities can be morphologically defined as pauci-nodular lesions with five or fewer small nodules or micronodules confined to a limited number of subsegmental secondary pulmonary lobules without overt cavities or tree-in-bud manifestations. Our provisional definition needs validation but will help investigate incipient TB on chest CT scans with uniform criteria. The number of studies seems too limited to suggest any provisional criteria for LN or metabolic abnormalities of incipient TB so far.

## Limitation of current understanding and future direction

A few limitations exist in the current radiologic and metabolic understanding of incipient TB. The sensitivity and specificity of chest CT and ^18^F-FDG PET scans for incipient TB. remain unclear yet. It is challenging to set a reference for assessing diagnostic accuracy: tools for confirming incipient TB are lacking, and minimal radiologic and metabolic abnormalities in TB contact investigations were typically regarded as active TB in the literature. Progression into active TB without treatment or regression by treatment may be indirect evidence for incipient TB, but few studies had an observation during follow-up. Furthermore, incipient TB. can naturally undergo a dynamic process without treatment, making the assessment of diagnostic accuracy more difficult.

The knowledge gap will be overcome by studies evaluating the diagnostic accuracy of advanced imaging modalities with predefined radiologic and metabolic criteria for incipient TB.. Prospective studies are also needed to examine the value of differing management based on a TB spectrum according to the presence of radiologic and metabolic abnormalities. More specifically, it is demanding to test whether adding to chest CT scans to the current contact investigation can help more effectively find active TB manifesting as normal radiographs in immunocompromised hosts and TB with minimal changes. Incipient TB. may have a higher chance for progression than latent TB but a higher chance for treatment success than active TB. In particular, it is crucial to systematically examine whether advanced imaging tools can prompt the treatment of drug-resistant TB at an earlier stage with a lower bacillary burden.

## Conclusions

Chest CT and ^18^F-FDG PET examinations in TB contact investigations can depict minimal pauci-nodular infiltrations in the lung parenchyma and metabolic uptake in the opacity and mediastinal LNs, in patients without any symptoms, X-ray abnormalities, or bacteriological/molecular evidence of active TB. Despite the paucity of follow-up data, human and nonhuman primate studies have found that such abnormalities may spontaneously regress, remain stagnant, or progress to active TB, and contacts with such abnormalities have a higher risk for progression than those without any abnormalities. In a contact investigation where the risk for TB infection and developing active TB is intense and active TB is more likely to manifest with normal radiographs, chest CT and ^18^F-FDG PET examinations may help further stratify contacts recently infected with *M. tuberculosis* along a continuous spectrum from latent tuberculosis to incipient, subclinical and active TB. In most contact investigations, a chest CT scan is preferable over an ^18^F-FDG PET scan, considering the accessibility, cost, and radiation dose. Identifying incipient TB would provide an opportunity for earlier and tailored treatment before active TB is established.

## Supplementary Information

**Additional file 1. Supplemental figure 1**. Preferred Reporting Items for Systematic Reviews and Meta-analyses diagram of the study selection process.** Supplemental Table 1**. Summary of definition for parenchymal and LN abnormalities in the included studies.

## Data Availability

Data sharing is not applicable to this article as no datasets were generated or analyzed in the current review.

## Abbreviations

18F-FDG: 18-Fluorodeoxyglucose
CT: Computed tomography
IGRA: Interferon-gamma release assay
LN: Lymph node
LTBI: Latent tuberculosis infections
MRI: Magnetic resonance imaging
Mtb: *M. tuberculosis*
PET: Positron emission tomography
TB: Tuberculosis
TST: Tuberculin skin test
