# Supplementary material for: CT and 18F-FDG PET abnormalities in contacts with recent tuberculosis infections but negative chest X-ray
Source: Insights Imaging. 2022 Jul 7;13:112. doi: 10.1186/s13244-022-01255-y (PMC9261169; doi:10.1186/s13244-022-01255-y)
Supplement: Supplementary file 1 — Additional file 1. Supplemental figure 1. Preferred Reporting Items for Systematic Reviews and Meta-analyses diagram of the study selection process. Supplemental Table 1. Summary of definition for parenchymal and LN abnormalities in the included studies. [file 13244_2022_1255_MOESM1_ESM.pdf]

<u>ELECTRONIC SUPPLEMENTARY MATERIAL</u>

CT and 18F-FDG PET abnormalities in contacts with recent tuberculosis infections but negative chest X-ray

Insights Imaging (2022) Yoon SH, Goo JM, Yim JJ, Yoshiyama T, Flynn JL

Supplemental figure 1. Preferred Reporting Items for Systematic Reviews and Meta-analyses diagram of the study selection process

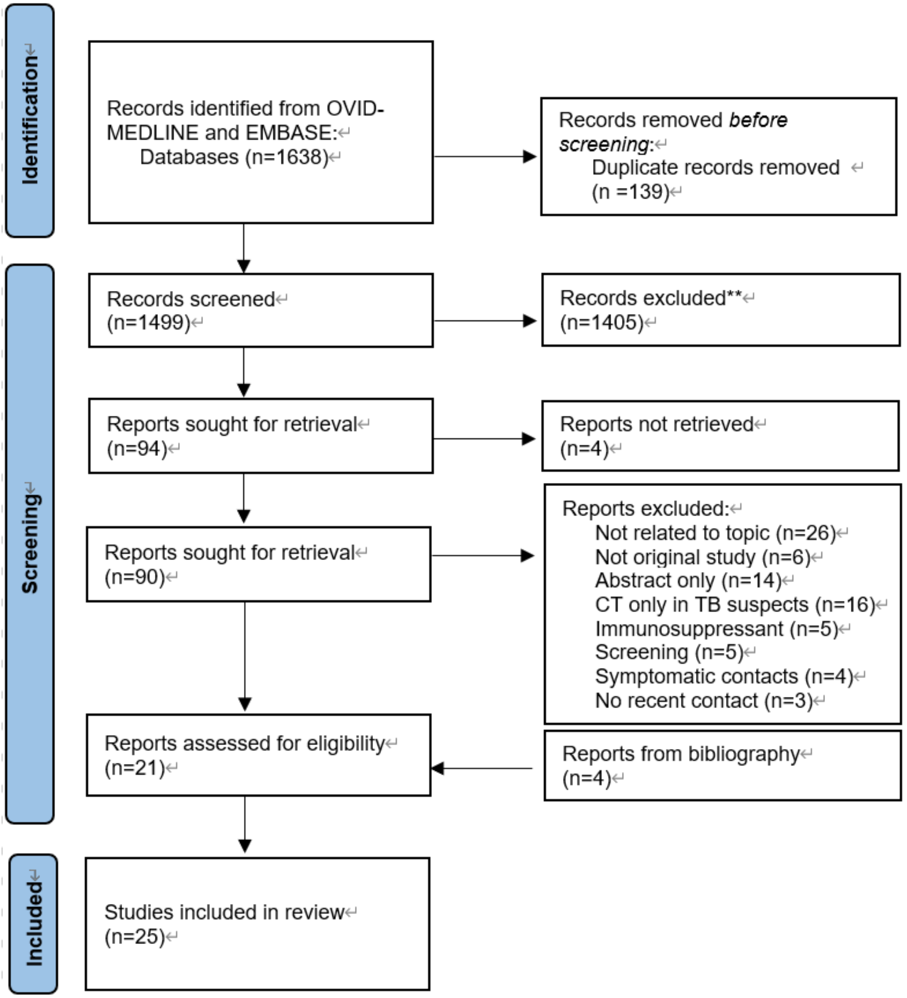

Insights Imaging (2022) Yoon SH, Goo JM, Yim JJ, Yoshiyama T, Flynn JL

Supplemental Table 1. Summary of definition for parenchymal and LN abnormalities in the included studies.

First author	Year	Country	Definition for parenchymal abnormalities*	Definition for LN abnormalities*
**Chest CT scan**				
Delacourt [13]	1993	France	Not specified	mediastinal LNs <4 years, 5 mm; 4-8 years 6 mm; >8 years, 7 mm; hilar LNs <4 years, 4mm; 4-8 years, 5 mm, >8 years, 6mm.
Duran [14]	1996	Spain	Not specified	mediastinal LNs <4 years, 5 mm; 4-8 years 6 mm; >8 years, 7 mm; hilar LNs <4 years, 4mm; 4-8 years, 5 mm, >8 years, 6mm.
Katakura [15]	1999	Japan	Not specified	Not specified
Baghaie [16]	2005	Iran	Not specified	Not specified
Yoshiyama [17]	2008	Japan	Not specified	Not specified
Lew [18]	2009	Korea	Not specified	Not specified
Lee [19]	2010	Korea	**The presence of cavities, branching linear opacity, or multiple noncalcified nodules	Not specified
Hirama [20]‡†	2011	Japan	Not specified	Not specified
Garrido [21]	2012	Spain	Not specified	Hilar or mediastinal LNs >10 mm
Fujikawa [22]	2014	Japan	**1) consolidation, 2) cavitation, 3) clusters of non-calcified nodules ≤4 mm associated with dilated or thickened peripheral airway walls, 4) non-calcified nodules >4 mm with adjacent small nodules, 5) widespread distribution of small nodules <4 mm	Not specified

Catho [23] ‡‡	2015	France	Not specified	Not specified
Lu [24]	2016	China	Not specified	Not specified
Ziemele [25]	2017	Latvia	Not specified	Not specified
Lee [26]‡‡†	2017	Korea	Not specified	Not specified
Shimizu [27]	2017	Japan	Not specified	Not specified
Moreno-Ballester [28]	2018	Spain	Not specified	Hilar or mediastinal LNs >5 mm
Yoshiyama [29]	2019	Japan	Not specified	Not specified
Zhou [30]	2019	China	Not specified	Not specified
Yoon [31] ‡‡‡	2020	Korea	Not specified	Not specified
Wang [32]	2020	China	Not specified	Not specified
Mok [33]	2021	Korea	Lesions that could not be clearly classified as active or fibrocalcified TB or lesions showing a few ill-defined centrilobular nodules or a non-calcified, indeterminate nodule on CT were classified as “indeterminate lesions.” A discrete and tiny nodule of <5mm in diameter by CT were considered “normal”	Not specified
**PET/CT scan**				
Ghesani [34]	2014	USA	A positive test was defined as FDG uptake greater than in the mediastinal blood pool, expressed as SUV. A true negative examination was defined as the absence of FDG uptake.	
Esmail [35]‡‡‡‡	2016	UK	Parenchymal lesions were categorized as infiltrates, fibrotic scars, active nodules or discrete nodules. Parenchymal lesions were considered to have abnormal if FDG uptake within lesion was > background lung parenchyma but less than that of mediastinal blood pool. Mediastinal and hilar lymph nodes was was considered abnormal if FDG uptake within lesion was < that of mediastinal blood pool but < that of liver	
**PET/MR scan**				
Molton [36]	2019	Singapore	A lymph node of > 1 cm in short axis was considered enlarged and a nodule > 6mm was considered significant.	

			Any lesion with standardized uptake value > 0.95 was considered abnormal	
Naftalin [37]	2020	Singapore	Not specified	Not specified

LN=lymph node; CT=computed tomography; ^{18}F-FDG=18-fluorodeoxyglucose; PET=positron emission tomography; MRI=magnetic resonance imaging; SUV=Standardized uptake unit; FDG=fluorodeoxyglucose.

*In studies without specifying definitions, the presence of subclinical abnormalities was evaluated based on radiologists interpretation

**Some studies provided the definition for active tuberculosis rather than subclinical abnormalities.
